# Supplementary material for: Nuclei-specific hypothalamus networks predict a dimensional marker of stress in humans
Source: Nat Commun. 2024 Mar 18;15:2426. doi: 10.1038/s41467-024-46275-y (PMC10948785; doi:10.1038/s41467-024-46275-y)
Supplement: Supplementary file 1 — Supplementary Information [file 41467_2024_46275_MOESM1_ESM.pdf]

Supplementary Information

Title: Nuclei-specific hypothalamus networks predict a dimensional marker of stress in humans

Authors

Daria EA Jensen, Klaus Ebmeier, Sana Suri, Matthew FS Rushworth, Miriam C Klein-Flügge

Supplementary Methods

Robustness of top edges

Several analyses depended on the ordering of the absolute regression weights extracted from the train group (**Figures 4A, 5C, 6C, Supplementary Figure 6D**), and on the weights estimated from the train group (**Figures 3C, 4A, 5C, 6C, Supplementary Figure 6D**). To test that results were robust to the precise procedure for extracting the top edges and the precise estimation of the weights, we compared two variations of computing them - both focused entirely on the train group: in the first variation, we fit one robust regression weight per edge using all n=198 train group participants; weights were then sorted according to their absolute regression coefficient. In the second variation, we performed n=10,000 internal splits of the n=198 train participants into two groups of n=99 participants. In each iteration, robust regressions were performed on half of the train group and the weights applied to the second half of the train group to predict stress scores internally within the train group. For predicting stress in the held-out test group (n=200), edges were then sorted according to the average correlation coefficient between predicted and real stress scores achieved across all 10,000 iterations for the held-out half of the train group. Weights used for test predictions in this second variation corresponded to the mean of all predictions of the held-out half of the train group obtained across the 10,000 iterations. Throughout the manuscript (**Figures 3C, 4A, 5C, 6C, Supplementary Figure 6D**), we show results using the first procedure, but all results hold using either procedure (see **Supplementary Results**).

Inclusion of additional hippocampus ROIs

Our a priori ROIs did not include the hippocampus. In an additional analysis, based on a reviewer suggestion, we included five subdivisions of the hippocampus as five additional ROIs. These were based on a parcellation by Tian et al.[1] and included: tail, body, head-l, head-m1, and head-m2. We computed the functional connectivity of these hippocampal subdivisions with each of the seven hypothalamus nuclei, leading to 5 x 7 = 35 additional edges. We then repeated the same analyses as before: (1) prediction of stress scores in the test group using all edges with weights estimated from the training group; (2) prediction of stress scores in the test group using iteratively more edges, sorted based on their importance in the training group, to find the peak and smallest significant network.

Supplementary Results

In **Figure 3C**, we showed that we could predict stress scores out-of-sample when regression coefficients were estimated based on the train group and applied to the functional connectivity of the test group. To test the robustness of these predictions, we examined if this prediction could withstand slight changes in the weights derived from the train group. As explained above, this time, instead of using the robust regression coefficients estimated from all n=198 train group participants, we generated n=10,000 splits of the train cohort into two halves (n=99 each). The test cohort was not touched during this step. In each of the 10,000 splits, for each edge, we computed the quality of the prediction obtained from a robust regression coefficient estimated on the first half of the train group when applied to the second half of the train group. The average prediction accuracy on the held-out half of the train data obtained for a given edge across the 10,000 splits was then used for the test group prediction. Using all 105 edges, the prediction of test group stress scores obtained using this second more robust approach looked virtually identical and again reached significance: the original approach predicted stress with *r*=0.265 (**Figure 3C**) and the second more robust approach with *r*=0.266 (**Supplementary Figure 5C**). In the analysis that added one connection at a time iteratively from 1 to 105, the top prediction was achieved with n=22 edges using the original approach (*r*=0.272; **Figure 4A**) and with n=57 edges using the second more robust approach (*r*=0.290; **Supplementary Figure 5C**). The earliest significant network for both approaches was identified with only one edge (SO/SC-RN-MR; *r*=0.130).

Looking at the order of edges identified with the two approaches shows that the first 15 connections are entirely the same with small differences in their ordering (100% overlap, see lists below); across the first 20 edges, the overlap is still 75%; from that point onwards, the ordering starts to differ slightly (60% overlap across the first 30 edges; non-overlapping edges are marked in grey).

The following are the top thirty connections in the order that resulted from the first variation (one fit on all n=198 train participants): SO/SC-RN-MR, MPO-amyg-Ce, PV-LC, PV-NAc, SO/SC-amyg-LaI, MPO-amyg-B, MPO-BNST, VM-LC, PV-amyg-Ce, SO/SC-amyg-LaD, PH-dPAG, DM-LC, PV-BNST, SO/SC-amyg-CoN, SO/SC-amyg-AB, SO/SC-SN, DM-amyg-LaV, DM-amyg-B, SO/SC-dPAG, VM-RN-MR, MM-amyg-B, PV-amyg-B, MM-amyg-CoN, PH-amyg-LaD, DM-dPAG, PV-amyg-AB, MPO-amyg-CoN, VM-amyg-Ce, MM-RN-MR.

These are the top 30 edges obtained from the second procedure (10,000 internal splits of the train group): SO/SC-RN-MR, MPO-amyg-Ce, PV-LC, SO/SC-amyg-LaI, PV-NAc, MPO-amyg-B, MPO-BNST, PV-amyg-Ce, PH-dPAG, VM-LC, DM-LC, SO/SC-amyg-LaD, PV-BNST, SO/SC-SN, SO/SC-amyg-AB, SO/SC-amyg-CoN, PV-amyg-B, MM-amyg-CoN, PH-RN-DR, VM-amyg-LaI, PH-SN, SO/SC-amyg-LaV, MM-RN-DR, MPO-NAc, SO/SC-amyg-B, MPO-SN, PH-vlPAG, PH-amyg-LaI, MM-vlPAG, VM_amyg_CoN (see first 22 top edges also in **Supplementary Table 5**).

We also repeated all analyses shown in **Figures 3-4**, using the 7T cohort instead of the 3T cohort as our test group (and all *n=398* 3T participants as our training group): comparison of the patterns of robust regression weights, out-of-sample prediction using all 105 edges, and predictions using increasing numbers of edges. Given the lack of variance in stress scores in this cohort, we expected out-of-sample predictions might prove challenging, and statistical tests were indeed non-significant (**Supplementary Figure 6**).

Finally, we repeated our key analyses, corresponding to **Figures 3C** and **4A** using 35 additional edges between five hippocampal subdivisions and our seven hypothalamus nuclei. We found that the inclusion of these additional edges did not improve the overall prediction of stress scores when using all predictors (r=0.157 instead of r=0.265). Similarly, when edges were included iteratively, adding the hippocampus to the set of ROIs did not further improve the predictions: the peak was not superior (updated peak prediction at 18 connections including hippocampus-to-hypothalamus: r=0.222 versus original peak prediction at 22 connections without hippocampus included: r=0.272). Nevertheless, among the strongest 20 predictors identified in the training set, we identified three edges between hypothalamus and hippocampus (VM to HClhead (5th), PV to HCm1head (11th), and VMa to HCtail (19th)). This suggests some hippocampus connections do indeed carry information relevant to stress. **Supplementary Figure 8** shows the results of these additional analyses.

Supplementary Discussion

Comparison of hypothalamus nuclei nomenclatures across existing parcellations

The naming of our clusters is based on the nomenclature of the clusters in the Atlas of the Human Brain[2], but sometimes it was necessary to group several nuclei together because of the coarser resolution of our data. The HCP resting-state data used here was acquired at a resolution of 2x2x2mm, which affected the separation of clusters using anatomical landmarks. For example, compared to Mai and colleagues[2] and other studies (e.g. [3]), we did not identify a lateral hypothalamus nucleus, likely because the spatial resolution in the medial-to-lateral plane was insufficient. We provide a detailed comparison of our parcellation with prior human hypothalamus subdivisions in **Supplementary Table 3** ([2–9]).

In the following, we will discuss how our parcellation compares with prior human hypothalamus subdivisions: Lechan & Toni[5] (see their Fig 12 and 23) reviewed the terminology of hypothalamic nuclei in the human brain and summarised nine main nuclei, which correspond to those used by Baroncini and colleagues[4] which were defined using T1/T2-weighted *in vivo* neuroimaging as well as a validation using post-mortem histology in a smaller sample. **Supplementary Table 3** shows our nomenclature side by side with these authors' terminology. Overall, there was good agreement between the parcellation of Mai et al.'s[2] and our parcellation, with their Pa nucleus corresponding to our PV nucleus, their PH agreeing with our PH, VMH with VM, and DMH with DM. In Lechan & Toni[5], our combined region SO/SC was referred to as the infundibular, SCh, SO and arcuate nucleus.

The comparison of our nuclei to the volumetric automatic *in vivo* MR parcellations by Billot and colleagues[6] and the manual MRI parcellation including post-mortem histological validation on n=2 subjects by Makris and colleagues[7] showed that our anterior nuclei PV and DM of the hypothalamus were grouped together into their anterior-superior nucleus, and that their anterior-inferior nucleus corresponded to our MPO. An equivalent nucleus to our PH was not present in their parcellation (compare with Billot and collegues[6], Fig 3). They also stated that the boundary between their anterior-inferior and anterior-superior nucleus was faint.

Ogawa and colleagues[8], using resting-state fMRI, defined seven comparable nuclei of the hypothalamus. Five nuclei agreed well, namely PV, DM, MPO, PH, and VM. The region

they referred to as arcuate nucleus was likely located within our MM, and their anterior nucleus matched our SO/SC well.

Neudorfer and colleagues[9] parcellation, which was performed on *in vivo* T1/T2-weighted structural MR images, overall agreed well with our parcellation – our PV agreed with their paraventricular and dorsal periventricular nuclei. DM, VM, PH and MPO were relatively comparable between parcellations. Neudorfer's[9] SO, SCh and AN likely correspond to our combined SO/SC, and our MM might be closest to their tuberomammillary nucleus. The key difference, where our and their parcellations did not agree well, was in terms of their large lateral nucleus. The lack of a clear separation between medial and lateral nuclei in our study made the comparison between this aspect of their parcellation and ours difficult. Using anatomical landmarks, we concluded that the lateral nucleus by Neudorfer and colleagues[9] might be partly included within our PV, MPO, SO/SC, DM and VM.

The parcellation of Schonknecht and colleagues[3] used in vivo diffusion tensor imaging (DTI) as well as T1/T2-weighted structural images. This parcellation was less detailed, delineating three subdivisions in lateral, anterior, and posteromedial hypothalamus.

In summary, our parcellation and the resulting boundaries between nuclei of the hypothalamus were largely consistent with the literature – in particular for PV, DM, VM, MPO, and PH. Other nuclei differed anatomically across different parcellations (e.g., SO/SC, MM). The anterior hypothalamus consists of SO/SC, but also contains the preoptic area including the MPO. SO/SC is sometimes also referred to as the infundibular or arcuate nucleus [5] or as the inferior tuberal nucleus[6]. The middle area of the hypothalamus consists of the PV, DM, and VM. We suggest that our PH is part of the posterior area of the hypothalamus. However, it may contain parts of the middle area. The posterior and mammillary body of the hypothalamus can be identified consistently across the literature and are contained in our MM nucleus.

Supplementary Tables

Supplementary Table 1: *Demographics of the three cohorts considered in this study consisting of n=200 3T-HCP participants (D1), n=200 non-overlapping 3T-HCP participants (R1) and n=98 non-overlapping 7T-HCP participants (R2):*

Mean ± SE, range	Original n=200 3T (D1)	Replication n=200 3T (R1)	Replication n=98 7T (R2)
**age**	28.95±.26, 22-36	28.28±.29, 22-36	29.42±.33, 23-36
**percentage of females**	54% (108)	49.5% (99)	60% (59)
**BMI**	26.33±.41, 0-44.7	25.83±.31, 17.48-41.76	26.65±.49, 19.22-4.27
**race**	.31±.06, 0-4	.325±.053, 0-4	.143±.044, 0-2
**ethnicity**	.095±.021, 0-1	.055±.016, 0-1	.03±.02, 0-1
**education***	14.98±.12, 11-17	15.09±.12, 11-17	14.74±.24, 0-17
**Total head motion**	.09±.002, .04-.27	.08±.002, .04-.2	.11±.01, .05-.31
**Intracranial volume x 100000**	15.87±.13, 1.83-19.93	15.95±.13, 9.45-2.43	15.82±.17, 11.41-19.32
**Total brain segmentation volume x 100000**	11.83±.08, 9.29-14.64	11.86±.09, 9.39-15.23	11.79±.12, 8.98-15.04

*Years of education completed: <11 = 11; 12; 13; 14; 15; 16; 17+ = 17

Supplementary Table 2: *Overview of human hypothalamus parcellations.*

Study	Sample	Parcellation modality		Procedure	Nuclei	Naming of nuclei
**Kullmann et al., 2014** [10]	n=49	3T	resting-state fMRI	automated	2	medial, lateral
**Piguet et al., 2011** [11]	Frontotemporal dementia (n = 18, validation: n = 12), healthy controls (n = 16, validation: n = 6)	3T	T1w	manual, validation using histology	2	anterior, posterior
**Schonknecht et al., 2013** [3]	n=10	3T	DTI T1w	automated and validation using T1w and T2w	3	anterior-medial, posterior-medial, lateral
**Baroncini et al., 2012** [4]	n=20, validation (n=6)	1.5T	T1w T2w	manual, validation using histology	4 (divided in 21 sub-nuclei)	preoptic, anterior, tuberal, posterior
**Goldstein et al., 2007** [12]	Schizophrenia cohorts (n = 88), relatives (n = 45), healthy controls (n = 48)	1.5T	T1w	manual	4	
**Spindler et al., 2020** [13]	Healthy (n=100) and validation (n = 20)	3T	DTI	automated	4	anterior-superior, anterior-inferior, intermediate, posterior
**Billot et al., 2020** [6]	Health controls and frontotemporal dementia (n = 37)	3T	T1w T2w	automated	5	anterior-superior anterior-inferior, superior-tuberal, inferior-tuberal, posterior
**Bocchetta et al., 2015** [14]	Health controls and frontotemporal dementia (each n = 18)	3T	T1w T2w	manual	5	
**Makris et al., 2013** [7]	n=44, validation (n=2)	7T and 1.5T	T1w	manual and validation using histology	5	
**Lemaire et al., 2011** [15]	Neurodegenerative disease (n = 7), Healthy controls (n = 7)	3T	DTI	manual	6	preoptic, supraoptic, anteroventral, anterodorsal, lateral, posterior
**Ogawa et al., 2020** [8]	n=400*	3T	resting-state fMRI	automated	7	anterior, arcuate, dorsomedial, medial preoptic, posterior, paraventricular
**Neudorfer et al., 2020** [9]	n = 900*, hypothalamus lesion patient (n = 1), deep brain stimulation patients (n = 2)	3T	T1w T2w	manual	13	anterior, arcuate, dorsal periventricular, dorsomedial, lateral, medial preoptic, paraventricular, periventricular, posterior, suprachiasmatic, supraoptic, tuberomammillary, ventromedial

* a subject mean template was used for the atlas generation

Supplementary Table 3: *Comparison of our nuclei nomenclature and nuclei labels in existing hypothalamus parcellations*

Our nuclei	Mai et al., 2016 [2]	Baroncini et al., 2012; Lechan & Toni, 2000 [4,5]	Billot et al., 2020; Makris et al., 2013 [6,7]	Ogawa et al., 2020 [8]	Neudorfer et al., 2020 [9]
**PV**	paraventricular nucleus (Pa + subdivisions)	paraventricular nucleus (Pa)	anterior-superior (a-sHyp)	paraventricular nucleus (PVH)	paraventricular nucleus (PA), dorsal periventricular nucleus (DP),
**MPO**	medial preoptic nucleus (MPO)	preoptic area (MPO)	anterior-inferior (a-iHyp)	medial preoptic nucleus (MPO)	medial preoptic nucleus (MPO), PE
**SO/SC**	supraoptic (SO), suprachiasmatic** (SChC and SChD)	infundibular or arcuate nucleus (Inf) supraoptic nucleus (SO), suprachiasmatic nucleus (SCh)	inferior tuberal (infTub)	anterior hypothalamic nucleus (AH)	Arcuate (AN) suprachiasmatic** (SCh), supraoptic (SO)
**DM**	dorsomedial nucleus (DM)	dorsomedial nucleus (DMH)	anterior-superior (a-sHyp)	dorsomedial nucleus (DMH)	dorsomedial nucleus (DM)
**VM**	ventromedial nucleus (VM)	ventromedial nucleus (VMH)	superior tuberal (supTub)	ventromedial nucleus (VMH)	ventromedial nucleus (VM), possibly AHA
**MM**	Inf, mammillary body (MM), intercalated nucleus	medial and mammillary nucleus (MM and LM)	posterior (posHyp)	arcuate nucleus (ARC)	tuberomammillary, lateral (TM)
**PH**	posterior (PH),	lateral (LHAp), posterior hypothalamic nucleus (PH),	--	posterior hypothalamic nucleus (PH)	posterior (PH),

* combined nuclei

**discussed as not present in our segmentation

Abbreviations for our parcellation: PV - paraventricular nucleus, MPO - medial preoptic nucleus, DM - dorsomedial nucleus, VM - ventromedial nucleus, PH - posterior hypothalamic nucleus, MM - mammillary body, SO/SC - supraoptic and suprachiasmatic nucleus.

Supplementary Table 4: *Overview of all ROIs used in this study. Each voxel has a size of 2x2x2 i.e., a volume of 8mm*^{3}.

ROI		Atlas	Size (voxels)	Reference
**Subcortical and Brainstem**	NAc	Harvard Subcortical Atlas	188	*Desikan et al., 2006; Frazier et al., 2005; Goldstein et al., 2007; Makris et al., 2006* [12,16–18]
	BNST		45	*Folloni et al., 2019* [19]
	SN	NITRC Atlas of the basal ganglia	134	*Keuken et al., 2014* [20]
	dPAG		45	*Faull et al., 2016* [21]
	vlPAG		43	
	dorsal RN	Harvard Ascending Arousal Network Atlas	23	*Edlow et al., 2012* [22]
	median RN		8	
	LC		20	*Betts et al., 2017* [23]
	Amygdala	FreeSurfer	647	*Glasser et al., 2013* [24]
**Amygdala nuclei**	Ce		62	*Klein-Flügge et al., 2022* [25]
	CoN		133	
	BAL		74	
	AB/BM		104	
	LaI		84	
	LaD		86	
	LaV/BL		104	
**Whole Hypothalamus**	Hypothalamus		109	*Zhou, 2017* [26]
**Total number of voxels** (% of all vertices)<br><br>**% of edges used out of all edges**			**1262** (1.38% of all 91,282 brain-ordinates)<br><br>**0.0015% edges**	

Abbreviations: HCP - Human Connectome Project, NITRC - NeuroImaging Tools and Resources Collaboratory, NAc - nucleus accumbens, BNST - bed nucleus of the stria terminalis, SN - substantia nigra, dPAG - dorsal periaqueductal grey, vlPAG - ventrolateral PAG, RN - raphe nuclei, LC - locus coeruleus, Ce - central amygdala nucleus, CoN - cortical amygdala nuclei, B - basal amygdala nucleus, AB/BM - auxiliary basal or basomedial amygdala nucleus, LaI - lateral intermediate amygdala nuclei, LaD - lateral dorsal amygdala nuclei, LaV/BL - lateral ventral portion containing portions of basolateral amygdala nucleus.

Supplementary Table 5: *Top 22 connections in the order included up until the peak in Figure 4A.*

number of edges	edges
1	SO/SC-RN-MR
2	MPO-amyg-Ce
3	PV-LC
4	PV-NAc,
5	SO/SC-amyg-LaI
6	MPO-amyg-B
7	MPO-BNST
8	VM-LC
9	PV-amyg-Ce
10	SO/SC-amyg-LaD
11	PH-dPAG
12	DM-LC
13	PV-BNST
14	SO/SC-amyg-CoN
15	SO/SC-amyg-AB
16	SO/SC-SN
17	SO/SC-amyg-LaV
18	DM-amyg-B
19	SO/SC-dPAG
20	VM-RN-MR
21	MM-amyg-B
22	PV-amyg-B

Abbreviations: PV - paraventricular nucleus, MPO - medial preoptic nucleus, DM - dorsomedial nucleus, VM - ventromedial nucleus, PH - posterior hypothalamic nucleus, MM - mammillary body, SO/SC - supraoptic and suprachiasmatic nucleus, NAc - nucleus accumbens, BNST - bed nucleus of the stria terminalis, SN - substantia nigra, dPAG - dorsal periaqueductal grey, RN - raphe nuclei, LC - locus coeruleus, amyg - amygdala, Ce - central amygdala nucleus, CoN - cortical amygdala nuclei, B - basal amygdala nucleus, AB - auxiliary basal or basomedial amygdala nucleus, LaI - lateral intermediate amygdala nuclei, LaD - lateral dorsal amygdala nuclei, LaV - lateral ventral portion containing portions of basolateral amygdala nucleus.

Supplementary Figures

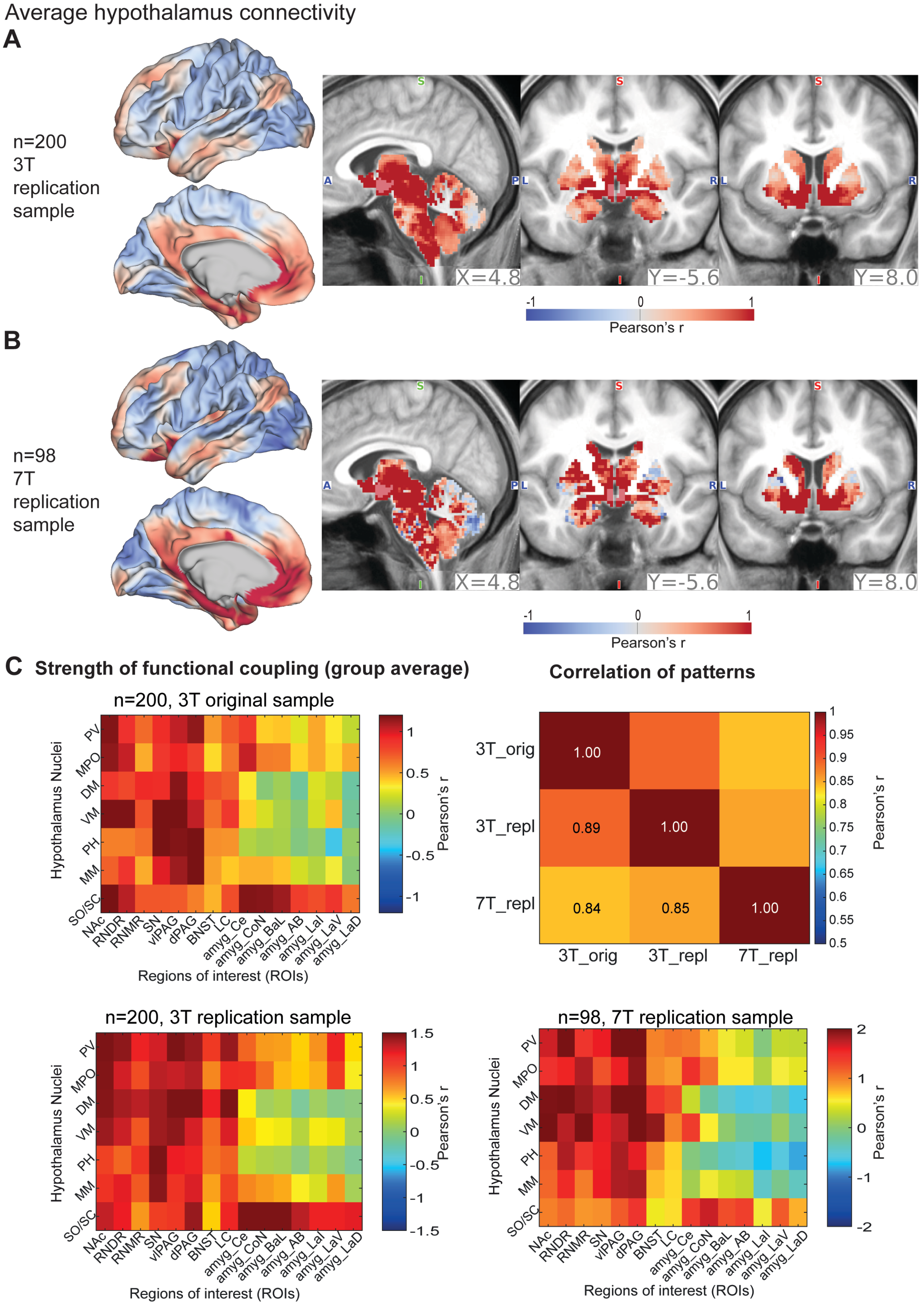

Supplementary Figure 1: Replication of group average hypothalamus functional connectivity.
A The group connectome shown in **Fig 1A** for n=200 3T participants of the young-adult HCP dataset was replicated for a second non-overlapping dataset of n=200 3T participants at the original voxel size of 2mm isotropic, and **B** for all remaining non-overlapping n=98 7T participants not included in either

3T datasets at a voxel resolution of 1.6mm isotropic. Colour scale indicates Pearson's correlation coefficient. Positive and negative functional connectivity is shown in red and blue colours, respectively. Hypothalamus outline is shown in semi-transparent colour; strong red values within the hypothalamus indicate strong autocorrelation of activity. Images were corrected for signal strength by dividing each brain ordinate's connectivity value by the brain ordinates' mean absolute connectivity to the whole brain. Note that unlike the 3T data, 7T data could not be corrected for breathing and pulsation artefacts during pre-processing because no physiological noise regressors were recorded. **C** Group average functional connectivity between hypothalamus nuclei and ROIs (in all cases using the original parcellation from n=200 3T dataset) shows strong similarity across cohorts, thus highlighting the robustness of the connectivity measures from hypothalamus nuclei, despite their small size. Top left: original n=200 3T sample (D1, as in Fig 1F); bottom left: n=200 3T replication sample (R1); bottom right: n=98 7T sample (R2). The correlation of the entire pattern across datasets shows high correlations between datasets. Scale bar denotes Pearson's r. Abbreviations: hypothalamus nuclei: PV, paraventricular; MPO, medial preoptic; DM, dorsomedial; VM, ventromedial; SO/SC, supraoptic and suprachiasmatic; PH, posterior; MM, mammillary bodies; regions of interest: substantia nigra (SN), bed nucleus of the stria terminalis (BNST), nucleus accumbens (NAc), dorsal and ventrolateral periaqueductal grey (dPAG/vlPAG), locus coeruleus (LC), dorsal and median raphe (RNDR, RNMR); amygdala nuclei: Ce - central, CoN - cortical, B - basal, AB/BM - auxiliary basal/basomedial, LaI - lateral intermediate, LaD - lateral dorsal, LaV/BL - lateral ventral/basolateral; A - anterior, P - posterior, S - superior, I - inferior, L - left, R - right.

A Evolution of hypothalamus cluster using hierarchical clustering in n=200 participants

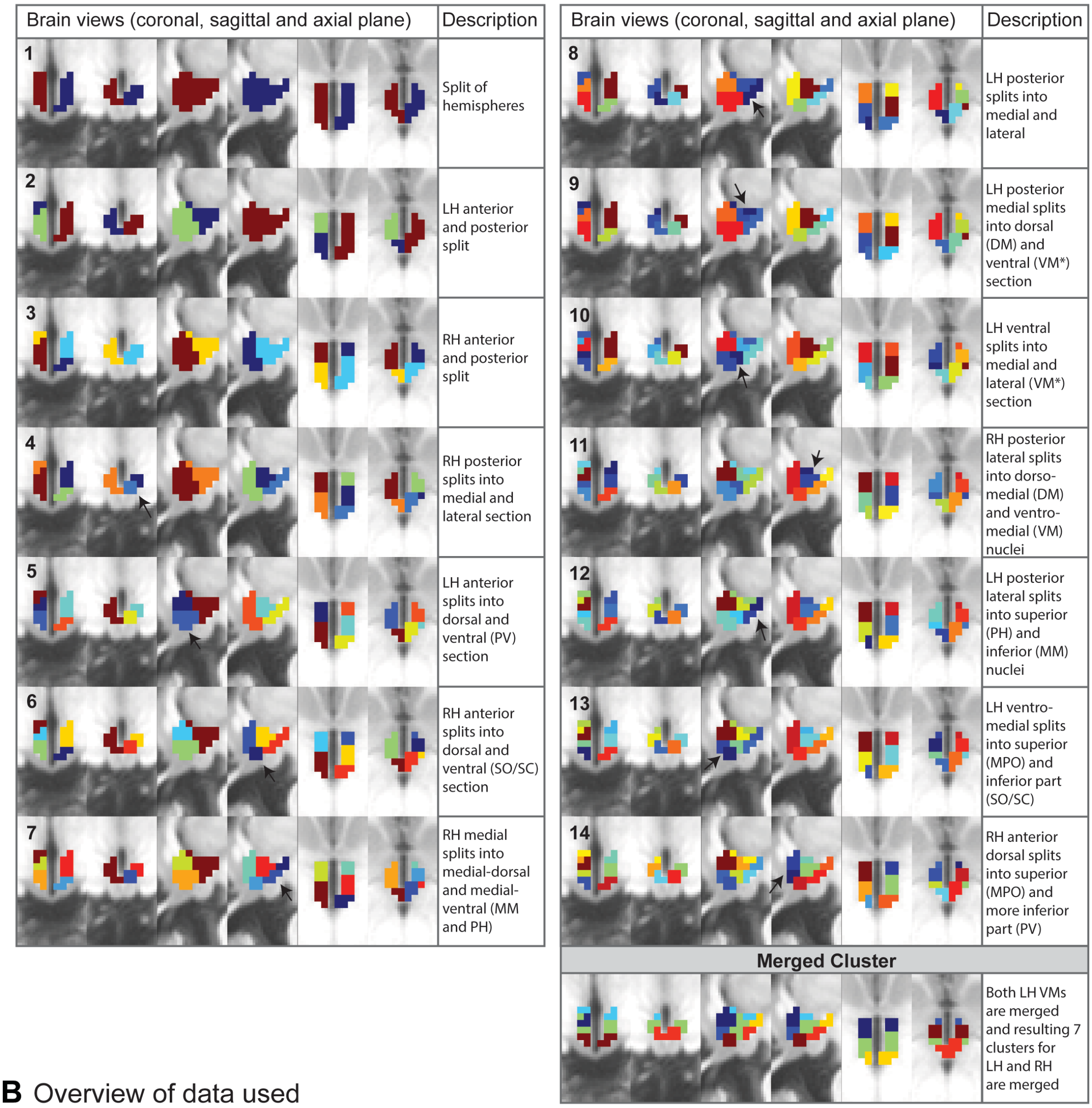

B Overview of data used

Parcellation of the hypothalamus

n=200	initial D1 (3T)	group connectivity and parcellation	Fig. 1, S2, S7, S9
n=200	replication R1 (3T)	replication group connectivity	Fig. S1
		replication parcellation	Fig. S3
n=98	replication R2 (7T)	replication group connectivity	Fig. S1
		replication parcellation	Fig. S3

Behaviour

n=400	dataset D1+R1 (3T)	behavioural factor analysis	Fig. 2
n=806	remaining dataset (3T)	replication behavioural factor analysis	Fig. S4
n=98	replication R2 (7T)	behavioural histogram plots	Fig. S4

Outlier rejection

Prediction (train ⟶ test)

n=200 (3T) ⟶	n=198 (3T)	Fig. 3-6, S5, S8
n=99 vs. n=99 (3T, robust prediction) ⟶	n=198 (3T)	Fig. S5
n=97 (7T) ⟶	n=398 (3T)	Fig. S6, S8

Supplementary Figure 2: Hypothalamus parcellation at different clustering depths and overview of used datasets.

A Hierarchical clustering was performed on the similarity between hypothalamus voxels in terms of their profile of absolute functional connectivity to the rest of the brain. Here we show how hypothalamus subdivisions evolved at different hierarchical clustering depths up until step 14, where bilateral clusters were combined, thus resulting in our final parcellation with a total of seven bilateral clusters. **B** Schematic overview of datasets used in this study.

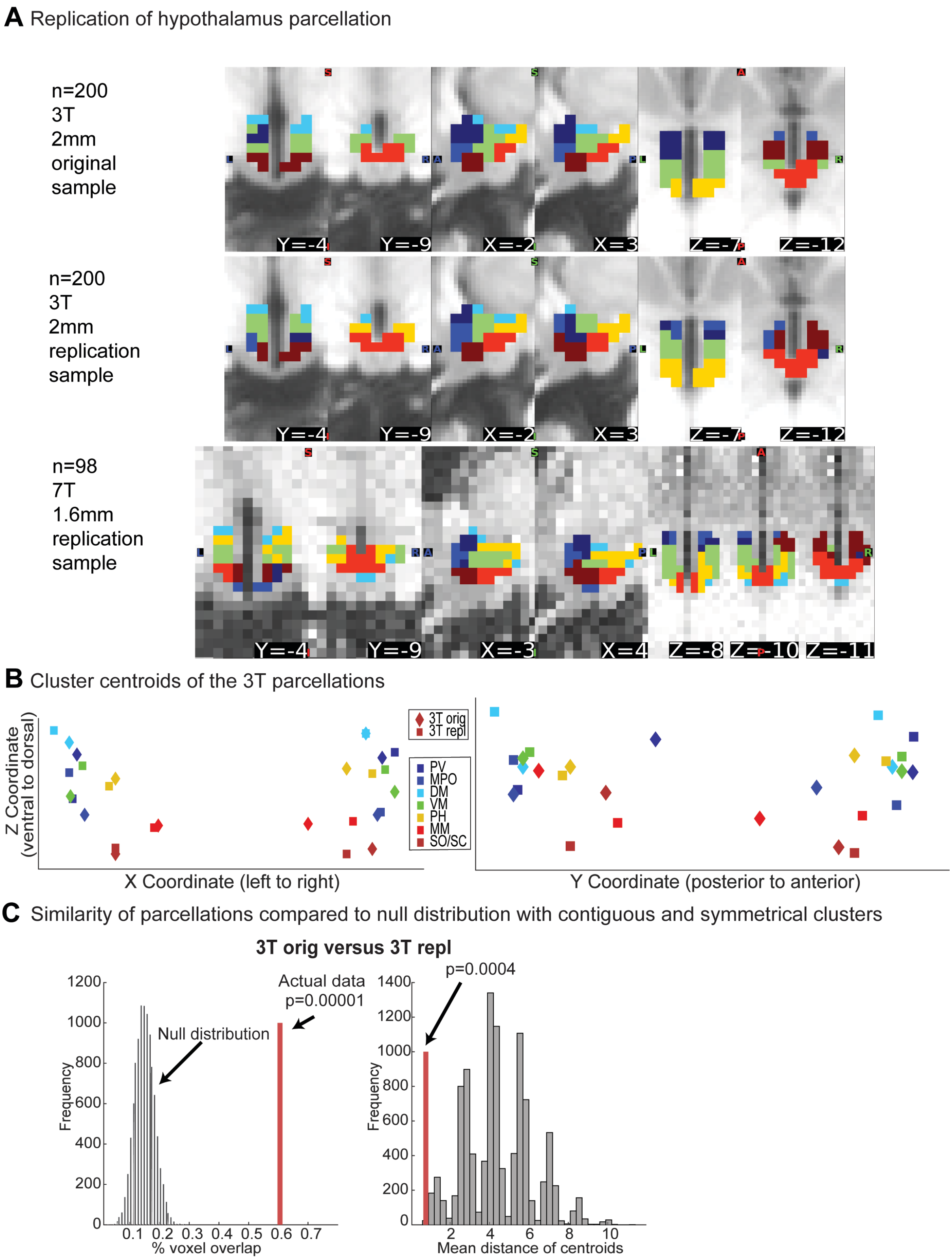

Supplementary Figure 3: Replication of hypothalamus parcellation.

A To validate the hypothalamus parcellation obtained using n=200 HCP participants and used throughout the study (top row, as in **Fig 1B, C**), we replicated the parcellation in two independent datasets: n=200 non-overlapping 3T participants (2mm isotropic voxels) and n=98 non-overlapping 7T participants (1.6mm isotropic voxels). The key subdivisions of the hypothalamus were replicated in both parcellations. **B** Illustration of the cluster centroids for the two 3T parcellations show close proximity of cluster centroids (left: coronal; right: sagittal). **C** The similarity of the two 3T parcellations

was evaluated statistically using two null distributions that quantified (a) the % of overlapping voxels expected by chance (voxels with identical labels) and (b) the mean distance of the centroids. The null distributions consider the size and symmetry of the original parcellation and shuffle the location of the nuclei so that they are non-overlapping and contiguous. The % overlap of 60.55% between the two 3T parcellations was higher than expected by chance (p=.00001) and the distance between centroids .7715 was smaller than expected by chance (p=.0004; one-sided p-values from nonparametric tests using permutation null distribution). Thus, the hypothalamus parcellation was robust across datasets. Note, however, that we use the original 3T parcellation across all analyses throughout the manuscript. In addition, this parcellation is based on the mean group connectivity and is thus orthogonal to key findings related to interindividual variation in stress. Source data for 3B are provided as a Source Data file.

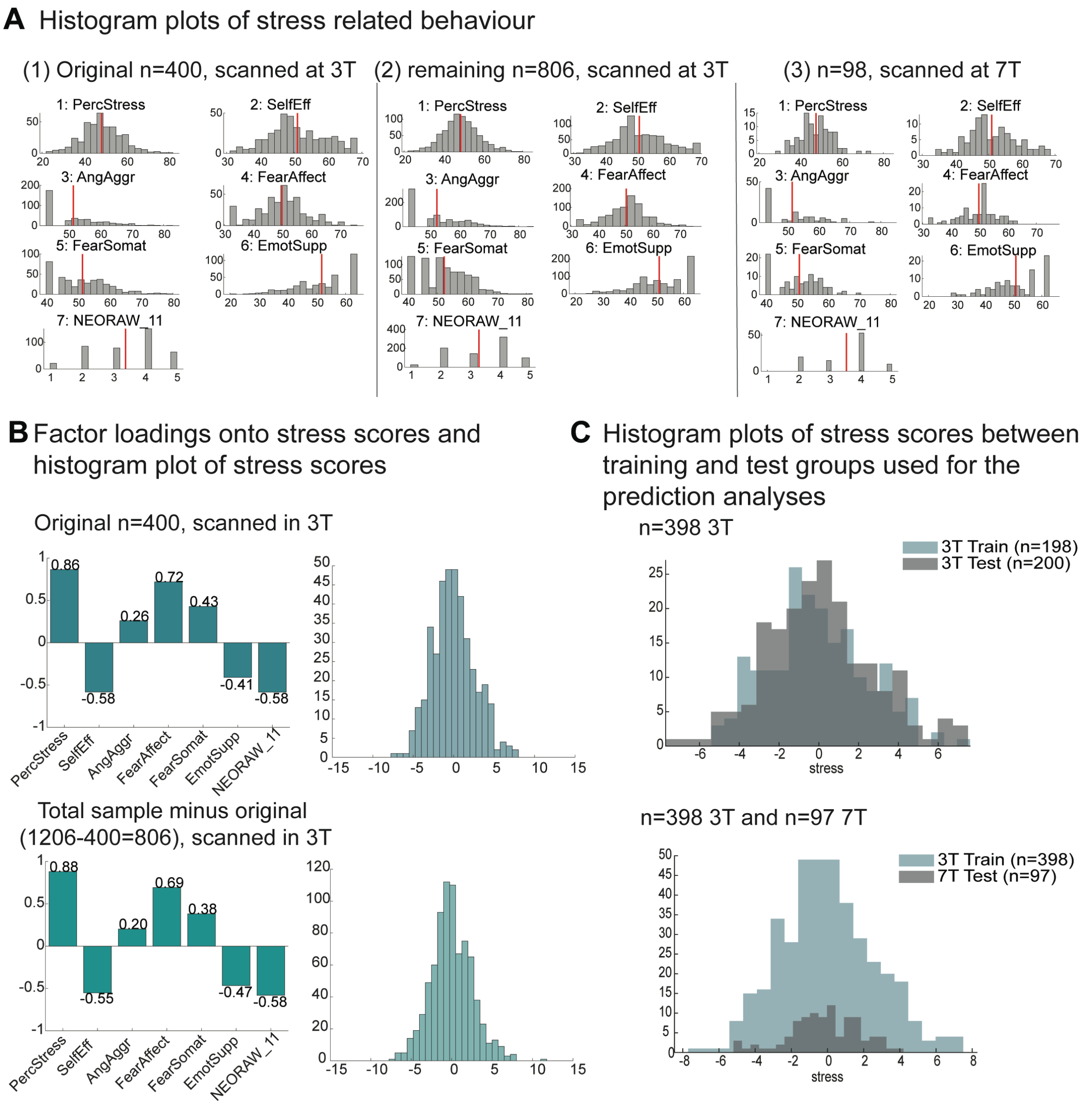

Supplementary Figure 4: Distribution and replication of factor analysis-derived dimensional stress score.

A Histograms of stress scores included in the factor analysis for (1) all 3T HCP participants included in this study, n=400 (before outlier rejection), (2) all remaining 3T HCP participants not included in this study (because of lacking resting-state data or physiological noise recordings); n=806 (n=1206-400), and (3) all 7T participants included in this study, n=98 (before outlier rejection). This shows lower variance in stress scores in the 7T cohort. **B** Factor loadings from the original factor analysis using n=400 3T participants (as in **Fig 2C**) replicate robustly when using a larger cohort of n=806 non-overlapping 3T participants, and the resulting dimensional stress scores are comparable. **C** For analyses presented in **Figs 3-6**, we split the n=400 3T participants into train and test cohorts such that stress scores were comparable (after outlier rejection: train n=198, test n=200). Accordingly, the histogram of factor-analysis derived dimensional stress scores for the 3T train and test groups shows comparable distributions (top). However, the variance in stress scores in the n=97 (after outlier rejection) 7T test group was considerably reduced compared to the full 3T cohort (n=398, bottom). The height of the histogram simply reflects differences in group size, but the reduced width of the histogram for 7T participants meant that analyses aiming to predict stress scores in the 7T cohort were hindered by this lack of variance.

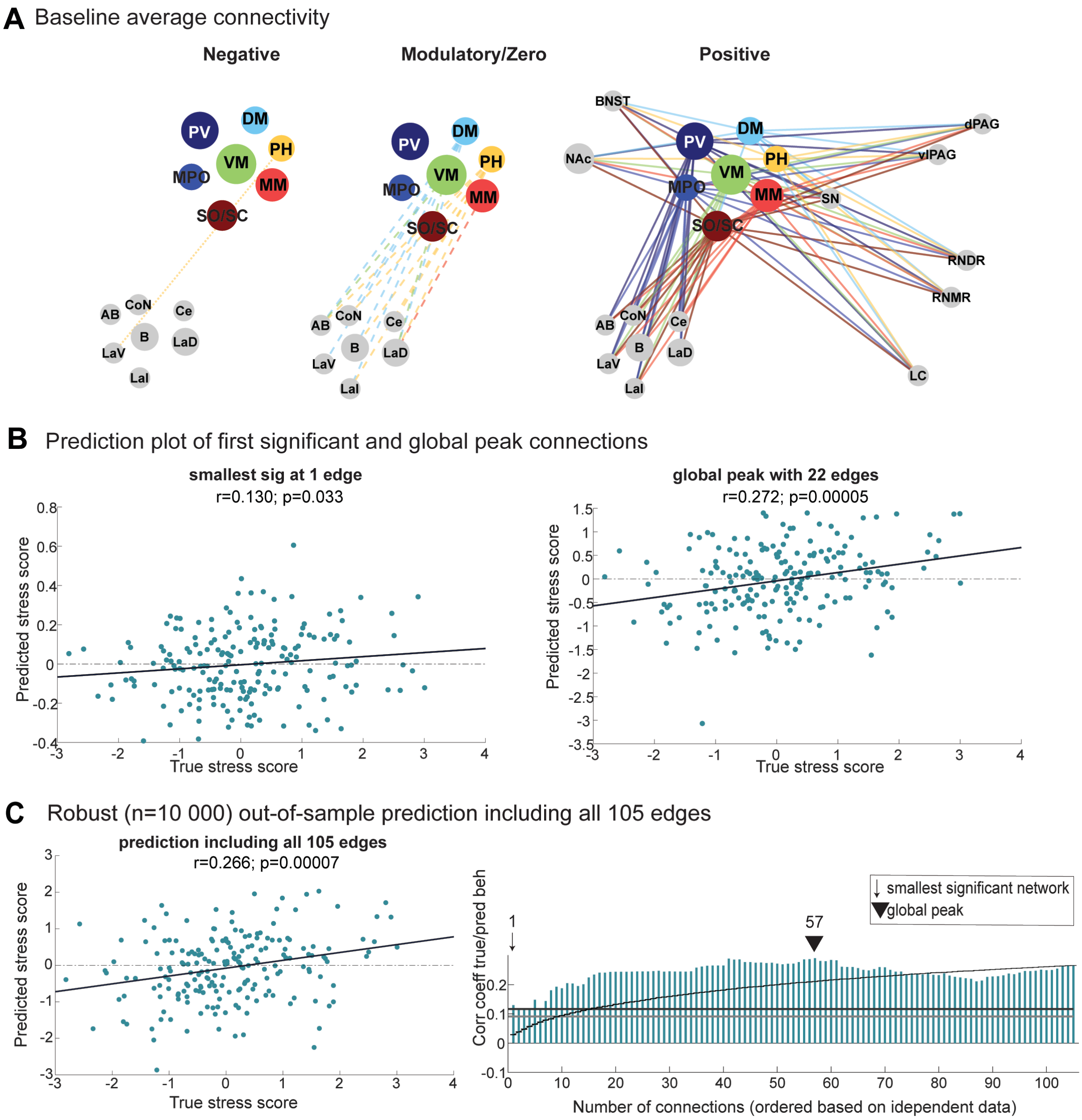

Supplementary Figure 5: Anatomical hypothalamus nuclei networks related to stress.

A Fingerprints are shown, independent of stress, for edges where group average functional connectivity was negative (left), modulatory/zero (middle), or positive (right) in the n=398 3T dataset (see also **Fig 1F, Supplementary Fig 1C**). Hypothalamus subcortical connectivity is on average largely positive, apart from a few edges between posterior-dorsal and -medial hypothalamus (PH, DM, VM) and amygdala where it is zero or negative. **B** Scatterplots show out-of-sample predictions of stress scores in the 3T test cohort generated using 3T train weights for the smallest significant (p<.05) network using one edge between SO/SC – RN-MR, as well as the peak prediction at n=22 edges (see also **Fig 4A**). **C** Additional validation of the weights and ordering of the edges used to predict stress in the test dataset: this time, the n=198 train dataset is split into two groups of n=99 each and this is repeated n=10,000 times. In each iteration, we use robust regressions to predict stress scores just internally within the train group (estimate weights on first n=99, predict stress scores in the second held-out n=99). The average r-value between predicted and true stress scores in the held-out half of the train group achieved across iterations is then used as weights to predict stress scores in the original n=200 fully held-out test participants. Using this approach, we again achieve a significant prediction of stress scores in the test group using the weights of the train group (left; r=.266, p=7×10^{-05}). Predictions of stress scores using smaller hypothalamus networks with subsets of edges between 1

and 105 (right) shows the smallest significant network emerges at one edge and the highest peak at 57 edges; plotting conventions as in **Fig 4A**. Edges were included in order of importance (absolute robust regression coefficients) estimated from the train group (n=198). Source data for 5A and C are provided as a Source Data file.

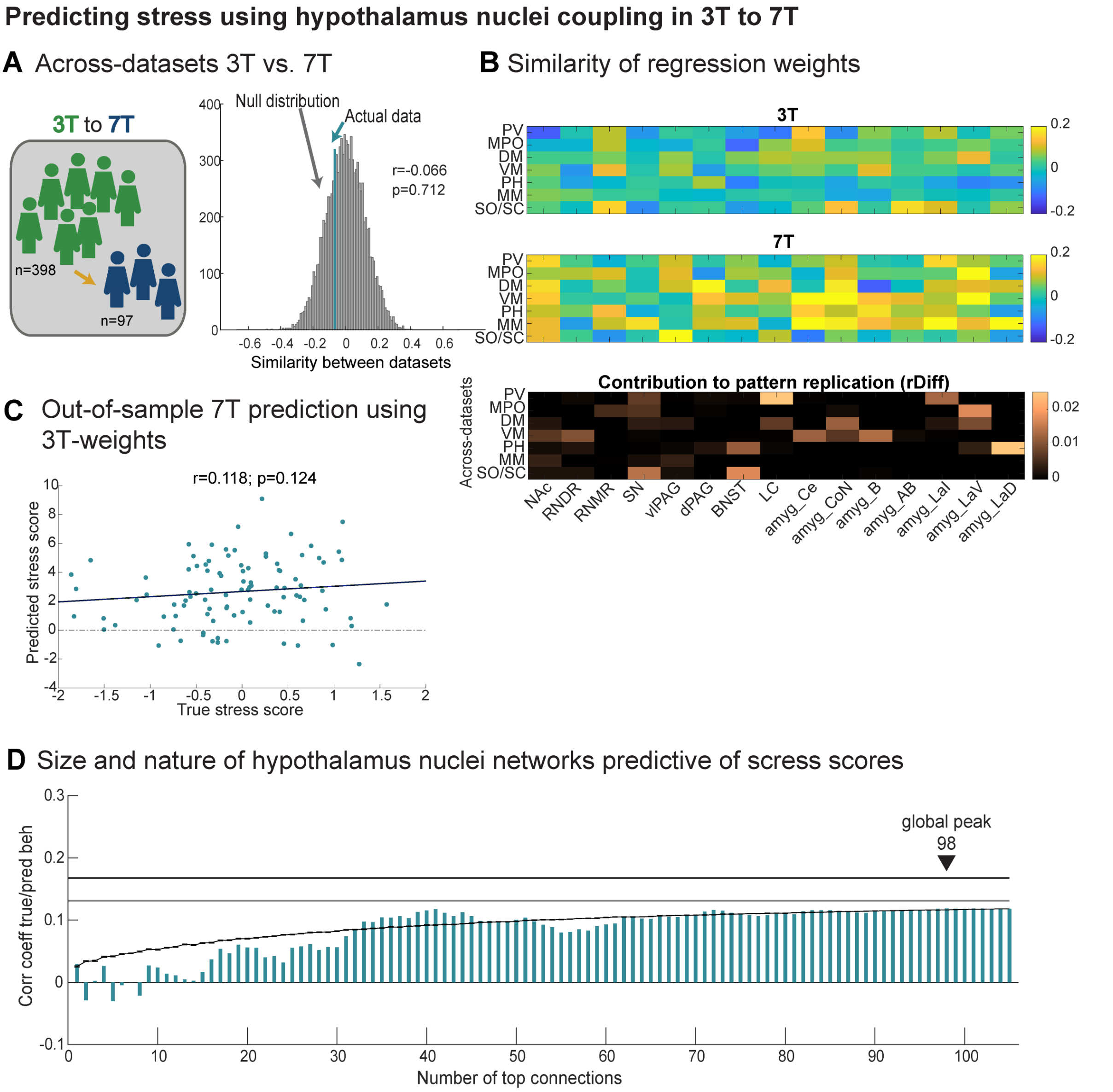

Supplementary Figure 6: Difficulties predicting stress scores in a cohort with reduced stress variance.

We attempted to replicate stress predictions using hypothalamus nuclei connectivity in a second held-out cohort, n=97 7T-HCP participants, using the full 3T cohort as our train data (n=398). We used identical procedures as in the 3T-to-3T replication reported in **Figs 3-4**. However, this time, predictions proved challenging, possibly due to reduced variance in stress scores (**Supplementary Fig 4C**). **A, B** The patterns of robust regression coefficients across datasets were not more similar than expected by chance (one-sided *P* values from non-parametric test using permutation null distributions). **c** An out-of-sample prediction of 7T stress scores generated based on 3T train weights did not reach significance ($r(95)$=.118; p=.124; CI=[-.08,.31]). **D** And predictions of stress scores using smaller hypothalamus networks with subsets of edges between 1 and 105 edges did not improve the predictions nor reach significance; plotting conventions as in **Fig 4A**. Source data for 6A-B and D are provided as a Source Data file.

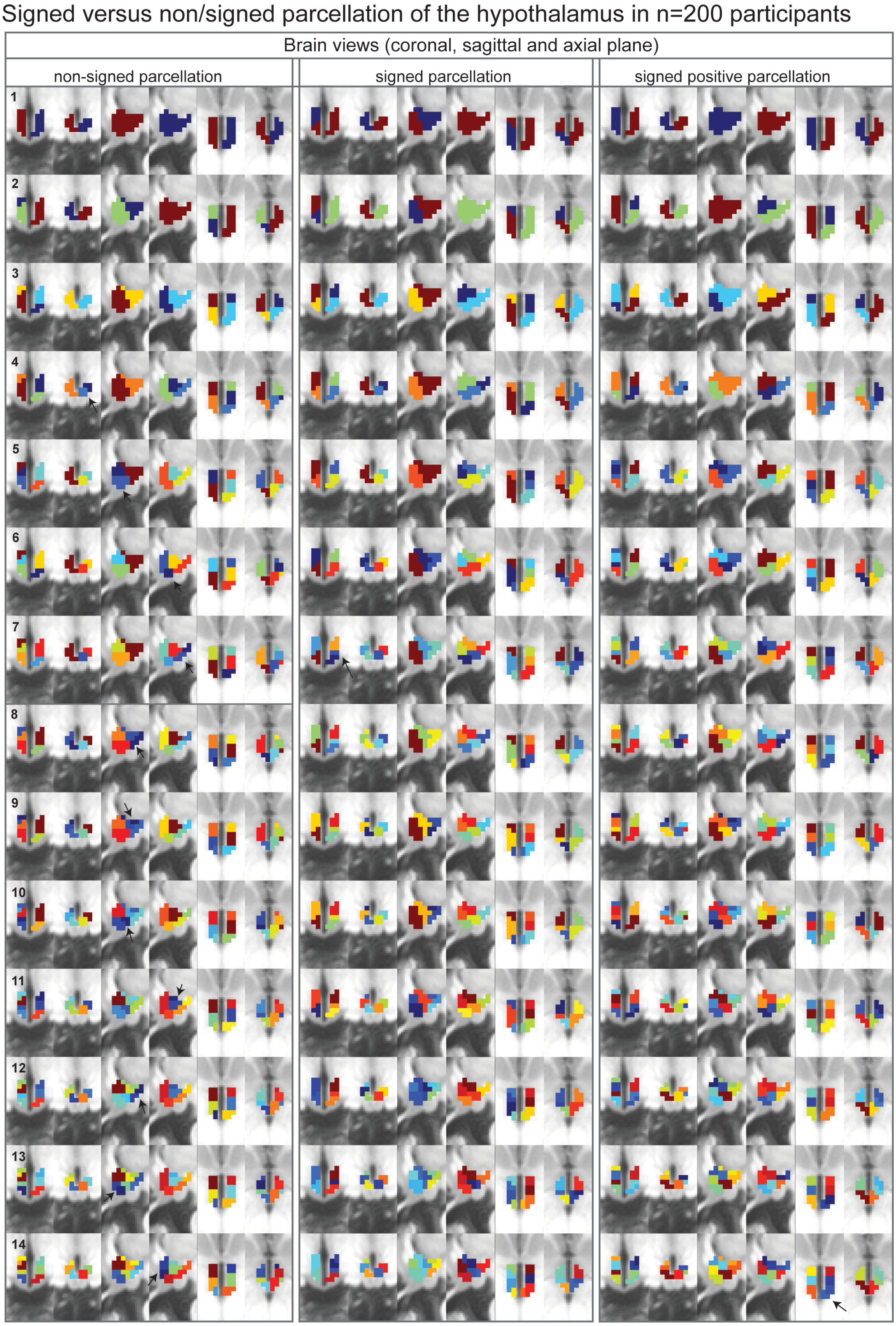

Supplementary Figure 7: Comparing hypothalamus parcellations from signed and absolute connectivity in n=200 participants.

Left: for the main parcellation presented in the manuscript ("non-signed" or absolute; left column), hierarchical clustering was performed on the similarity between hypothalamus voxels in terms of their profile of absolute functional connectivity to the rest of the brain. When using absolute connectivity values, correlation coefficients in the similarity matrix will be driven by strong versus weak connectivity differences. Here we show how hypothalamus subdivisions evolved at different hierarchical clustering depths up until step 14. By contrast, for the "signed" parcellation (middle column), clustering was performed on the similarity matrix derived from both positive and negative functional connectivity. This means that correlation coefficients in the similarity matrix will be driven by negative versus positive connectivity differences of the hypothalamus with the rest of the brain. In the "signed positive" column, the parcellation was performed on a similarity matrix computed using only positive functional coupling values, i.e., excluding any regions with negative functional coupling with the hypothalamus.

Predicting stress using hypothalamus nuclei coupling with hippocampal sub-groups

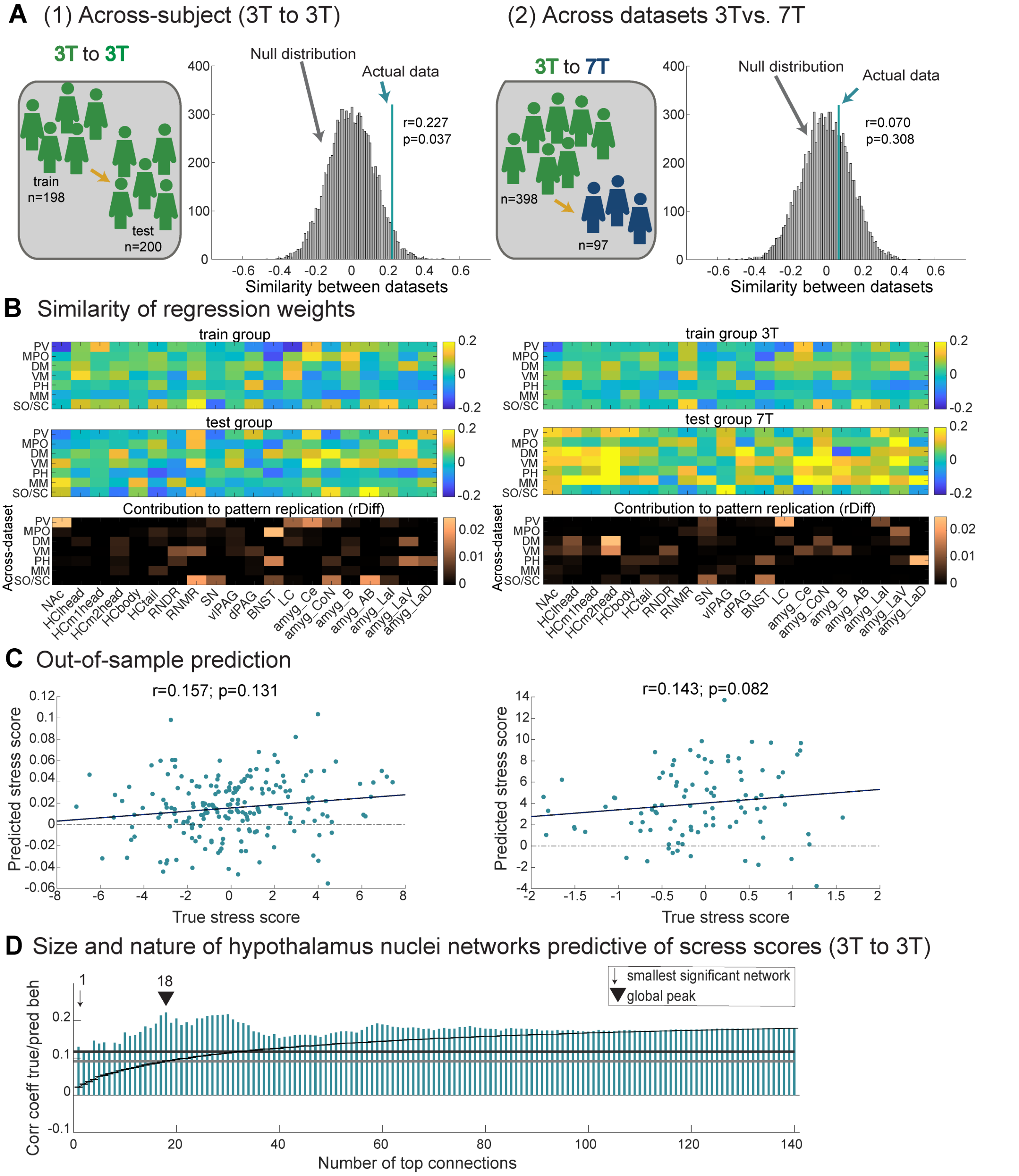

Supplementary Figure 8: Predicting stress scores using hypothalamus nuclei coupling with five additional hippocampal subregions.

We replicated stress predictions with an additional five hippocampal subregions from[1]. Instead of 105 edges (7 hypothalamus nuclei x 15 ROIs), we included 140 edges between 7 hypothalamus nuclei and 20 ROIs. **A** The pattern of regression coefficients in B were more similar than expected by chance. This was true (1) across two 3T datasets (left), but not (2) across datasets (right). **B** Visualization of robust regression coefficients capturing relationships with stress illustrates the similarity of the patterns statistically evaluated in (a). The contribution of each edge was calculated using the difference in correlation coefficient when excluding this edge versus including all edges (rDiff, bottom row). Visual inspection of rDiff values highlights strong similarities between (1) but not (2). Overall, hippocampus connections do not seem to contribute to stress predictions, apart from hHCm2head to DM and VM.

C Regression coefficients estimated from the train group on all 140 edges were applied to the functional connectivity of the test group to calculate predicted stress scores. This showed non-significant out-of-sample predictions of true stress scores, suggesting addition of all hippocampus subregions worsened predictions (compare with main Fig 3C). **D** Prediction of stress scores obtained using between 1 and 140 edges including hippocampus subregions. Edges were included in order of absolute robust regression coefficients from the train group (n=198). Prediction (turquoise bars) are shown as the correlation between true and predicted stress scores in the test participants (n=200), but were only statistically evaluated at the peak (black triangle: 'global peak') and to derive the smallest number of edges that reached a significant out-of-sample prediction (black arrow, 'smallest significant network'); black curve indicates performance using same number of edges included in random order; black line indicates threshold for significance at P<.05 for visualisation (grey line: P<0.1, r=.0911). A significant prediction could be achieved using only the first edge (SO/SC to RNMR; r=.131). The best prediction was achieved using 18 edges (r=.222), however again this prediction was lower than that achieved without inclusion of the additional hippocampus regions (compare with main Fig 4A). Source data for 8B and D are provided as a Source Data file.

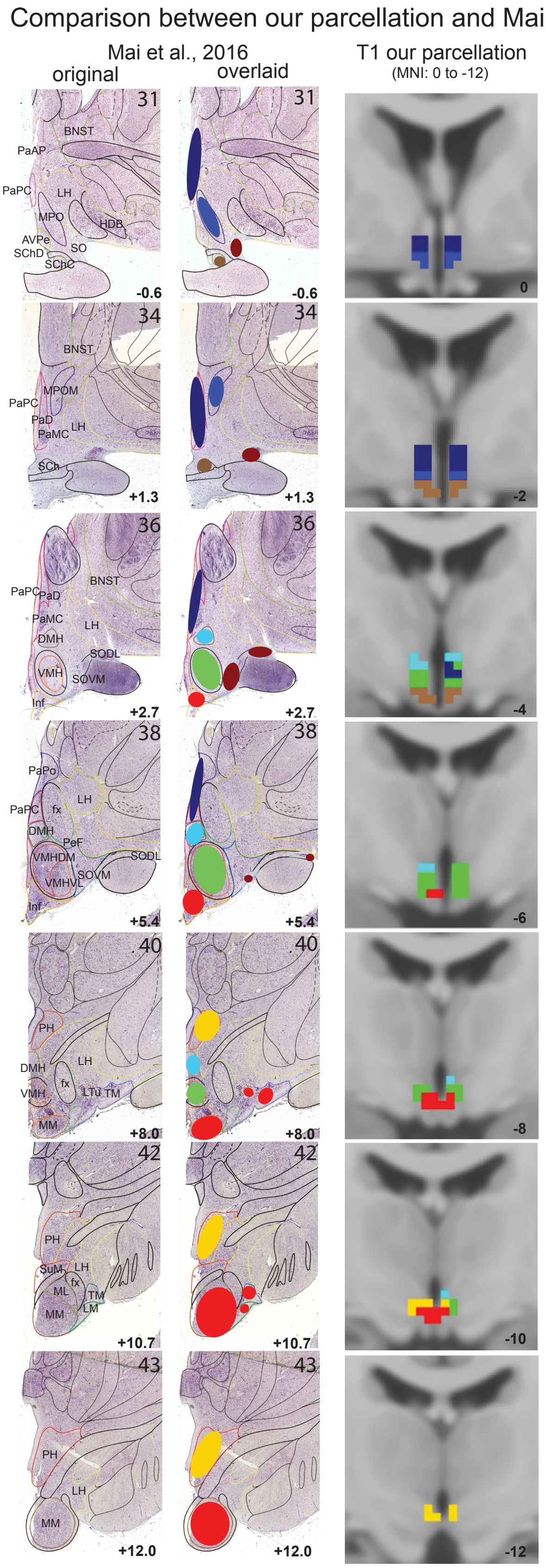

Supplementary Figure 9: Side-by-side comparison with hypothalamus atlas.

Comparison between our parcellation and hypothalamus nuclei in the Atlas of the human brain in Mai et al., (2016). The coronal sections of Mai et al. (2016; left) were overlaid with our identified nuclei (middle) which are again shown on the right. Our nuclei are indicated as follows: PV - paraventricular

nucleus (dark blue), MPO - medial preoptic nucleus (middle blue), DM - dorsomedial nucleus (light blue), VM - ventromedial nucleus (green), PH - posterior hypothalamic nucleus (yellow), MM - mammillary body (red), SO/SC - supraoptic and suprachiasmatic nucleus (dark red and brown). Atlas sections (left and middle column) from the Atlas of the human brain were used to facilitate the delineation of hypothalamic and surrounding structures: AVPe - anteroventral periventricular hypothalamic nucleus; BNST - bed nucleus of stria terminalis; DMH - dorsomedial hypothalamic nucleus; fx - fornix; HDB - horizontal limb of the diagonal band; LH - lateral hypothalamus; ML - medial mammillary nucleus - lateral part; MM - mammillary bodies; MPO - medial preoptic nucleus (includes MPO, MPOM); PA - paraventricular nucleus (includes PaAP, PaPC, PaMC, PaPo and PaD); PH - posterior hypothalamus; SCh - suprachiasmatic nucleus (includes SChD, SCHC); SO - supraoptic nucleus (includes SO, SOVM, SODL); SuM - supramammillary nucleus; TM - tuberomammillary nucleus; VMH - ventromedial nucleus (includes VMH, VMHVL, VMHDM). Reproduced with permission from Mai JK, Paxinos G, Voss T (2016): Atlas of the Human Brain, 4th ed. San Diego: Elsevier Academic Press. Source data for our hypothalamus nuclei are provided as a Source Data file.

Supplementary References

1. Tian, Y., Margulies, D. S., Breakspear, M. & Zalesky, A. Topographic organization of the human subcortex unveiled with functional connectivity gradients. *Nat. Neurosci.* **23**, 1421–1432 (2020).
2. Mai, J., Majtanik, M, & Paxinos, G. *Atlas of the Human Brain*. vol. 4th edition (Academic press, 2016).
3. Schonknecht, P. *et al.* Diffusion imaging-based subdivision of the human hypothalamus: a magnetic resonance study with clinical implications. *Eur. Arch. Psychiatry Clin. Neurosci.* **263**, 497–508 (2013).
4. Baroncini, M. *et al.* MRI atlas of the human hypothalamus. *NeuroImage* **59**, 168–180 (2012).
5. Lechan, R. M. & Toni, R. *Functional Anatomy of the Hypothalamus and Pituitary*. *Endotext* (MDText.com, Inc., 2000).
6. Billot, B. *et al.* Automated segmentation of the hypothalamus and associated subunits in brain MRI. *NeuroImage* **223**, 117287 (2020).
7. Makris, N. *et al.* Volumetric parcellation methodology of the human hypothalamus in neuroimaging: normative data and sex differences. *NeuroImage* **69**, 1–10 (2013).
8. Ogawa, A. *et al.* Connectivity-based localization of human hypothalamic nuclei in functional images of standard voxel size. *NeuroImage* **221**, 117205 (2020).
9. Neudorfer, C. *et al.* A high-resolution in vivo magnetic resonance imaging atlas of the human hypothalamic region. *Sci. Data* **7**, 305 (2020).
10. Kullmann, S. *et al.* Resting-state functional connectivity of the human hypothalamus. *Hum. Brain Mapp.* **35**, 6088–6096 (2014).
11. Piguet, O. *et al.* Eating and hypothalamus changes in behavioral-variant frontotemporal dementia. *Ann. Neurol.* **69**, 312–319 (2011).
12. Goldstein, J. M. *et al.* Hypothalamic abnormalities in schizophrenia: sex effects and genetic vulnerability. *Biol. Psychiatry* **61**, 935–945 (2007).
13. Spindler, M., Özyurt, J. & Thiel, C. M. Automated diffusion-based parcellation of the hypothalamus reveals subunit-specific associations with obesity. *Sci. Rep.* **10**, 22238 (2020).
14. Bocchetta, M. *et al.* Detailed volumetric analysis of the hypothalamus in behavioral variant frontotemporal dementia. *J. Neurol.* **262**, 2635 (2015).
15. Lemaire, J.-J. *et al.* White matter connectivity of human hypothalamus. *Brain Res.* **1371**, 43–64 (2011).
16. Desikan, R. S. *et al.* An automated labeling system for subdividing the human cerebral cortex on MRI scans into gyral based regions of interest. *NeuroImage* **31**, 968–980 (2006).
17. Frazier, J. A. *et al.* Structural brain magnetic resonance imaging of limbic and thalamic volumes in pediatric bipolar disorder. *Am. J. Psychiatry* **162**, 1256–1265 (2005).
18. Makris, N. *et al.* Decreased volume of left and total anterior insular lobule in schizophrenia. *Schizophr. Res.* **83**, 155–171 (2006).
19. Folloni, D. *et al.* Dichotomous organization of amygdala/temporal-prefrontal bundles in both humans and monkeys. *eLife* **8**, e47175 (2019).
20. Keuken, M. C. *et al.* Quantifying inter-individual anatomical variability in the subcortex using 7 T structural MRI. *NeuroImage* **94**, 40–46 (2014).
21. Faull, O. K., Jenkinson, M., Ezra, M. & Pattinson, K. T. Conditioned respiratory threat in the subdivisions of the human periaqueductal gray. *eLife* **5**, e12047 (2016).

22. Edlow, B. L. *et al.* Neuroanatomic connectivity of the human ascending arousal system critical to consciousness and its disorders. *J. Neuropathol. Exp. Neurol.* **71**, 531–546 (2012).
23. Betts, M. J., Cardenas-Blanco, A., Kanowski, M., Jessen, F. & Düzel, E. In vivo MRI assessment of the human locus coeruleus along its rostrocaudal extent in young and older adults. *NeuroImage* **163**, 150–159 (2017).
24. Glasser, M. F. *et al.* The minimal preprocessing pipelines for the Human Connectome Project. *NeuroImage* **80**, 105–124 (2013).
25. Klein-Flügge, M. *et al.* Relationship between nuclei-specific amygdala connectivity and mental health dimensions in humans. *Nat Hum Beh* (2022).
26. Zhou, Y. Abnormal structural and functional hypothalamic connectivity in mild traumatic brain injury. *J. Magn. Reson. Imaging* **45**, 1105–1112 (2017).
