# Supplementary material for: Nuclei-specific hypothalamus networks predict a dimensional marker of stress in humans
Source: Nat Commun. 2024 Mar 18;15:2426. doi: 10.1038/s41467-024-46275-y (PMC10948785; doi:10.1038/s41467-024-46275-y)
Supplement: Supplementary file 2 — Reporting Summary [file 41467_2024_46275_MOESM2_ESM.pdf]

nature portfolio

Corresponding author(s): Daria Jensen and Miriam Klein-Flügge

Last updated by author(s): Jan 27, 2024

Reporting Summary

Nature Portfolio wishes to improve the reproducibility of the work that we publish. This form provides structure for consistency and transparency in reporting. For further information on Nature Portfolio policies, see our Editorial Policies and the Editorial Policy Checklist.

Statistics

For all statistical analyses, confirm that the following items are present in the figure legend, table legend, main text, or Methods section.

n/a	Confirmed	
☐	☒	The exact sample size (*n*) for each experimental group/condition, given as a discrete number and unit of measurement
☐	☒	A statement on whether measurements were taken from distinct samples or whether the same sample was measured repeatedly
☐	☒	The statistical test(s) used AND whether they are one- or two-sided<br>*Only common tests should be described solely by name; describe more complex techniques in the Methods section.*
☐	☒	A description of all covariates tested
☐	☒	A description of any assumptions or corrections, such as tests of normality and adjustment for multiple comparisons
☐	☒	A full description of the statistical parameters including central tendency (e.g. means) or other basic estimates (e.g. regression coefficient) AND variation (e.g. standard deviation) or associated estimates of uncertainty (e.g. confidence intervals)
☐	☒	For null hypothesis testing, the test statistic (e.g. *F*, *t*, *r*) with confidence intervals, effect sizes, degrees of freedom and *P* value noted<br>*Give P values as exact values whenever suitable.*
☒	☐	For Bayesian analysis, information on the choice of priors and Markov chain Monte Carlo settings
☒	☐	For hierarchical and complex designs, identification of the appropriate level for tests and full reporting of outcomes
☐	☒	Estimates of effect sizes (e.g. Cohen's *d*, Pearson's *r*), indicating how they were calculated

Our web collection on statistics for biologists contains articles on many of the points above.

Software and code

Policy information about availability of computer code

Data collection	Since the data used in this manuscript were provided by the Human Connectome Project (https://www.humanconnectome.org/), no software was used for data collection purposes.
Data analysis	We used minimally preprocessed data from the Human Connectome Project (for details, see Methods and Klein-Fluegge, Nat Hum Beh, 2022). Further data processing and analysis was performed using tools from FSL (version 6.0; PALM (https://fsl.fmrib.ox.ac.uk/fsl/fslwiki/PALM), PNM toolbox (https://fsl.fmrib.ox.ac.uk/fsl/fslwiki/PNM) , FSLnets (https://fsl.fmrib.ox.ac.uk/fsl/fslwiki/FSLNets)), Freesurfer (version 5.2), MIGP for group-PCA (Smith et al., NeuroImage, 2014), Connectome Workbench (version 1.3.2), command-line functions and custom-written MATLAB (R2021a), R (version 4.2.1 in RStudio version 2022.12.0+353) and BASH scripts. OSF repository for the analysis pipeline used: osf.io/bq3fd.

For manuscripts utilizing custom algorithms or software that are central to the research but not yet described in published literature, software must be made available to editors and reviewers. We strongly encourage code deposition in a community repository (e.g. GitHub). See the Nature Portfolio guidelines for submitting code & software for further information.

Data

Policy information about availability of data

All manuscripts must include a data availability statement. This statement should provide the following information, where applicable:

- Accession codes, unique identifiers, or web links for publicly available datasets
- A description of any restrictions on data availability
- For clinical datasets or third party data, please ensure that the statement adheres to our policy

All data used in the present study are available for download from the Human Connectome Project (www.humanconnectome.org). Users must apply for access and agree to the HCP data use terms (for details see https://www.humanconnectome.org/study/hcp-young-adult/data-use-terms). Here we used both Open Access and Restricted data. Masks of all ROIs used and of the individual hypothalamus nuclei generated in this study are available in the OSF repository: osf.io/bq3fd. CCC licence is provided for hypothalamus images used in Supplementary Figure 9 from by 'The Atlas of the Human Brain' by Mai et al., (2016, http://atlas.thehumanbrain.info/).

Research involving human participants, their data, or biological material

Policy information about studies with human participants or human data. See also policy information about sex, gender (identity/presentation), and sexual orientation and race, ethnicity and racism.

Reporting on sex and gender	Sex (self reported) was used in this study.<br>Datasets: In D1 (3T, n=200), we included 108 females; In R1 (3T, n=200), we included 99 females; In R2 (7T, n=98), we included 59 females.
Reporting on race, ethnicity, or other socially relevant groupings	An overview of reported demographic variables can be found in Supplementary Table 1. In this study we used:<br>race (Self reported, race was classified as: White, Black or African American, and Other)<br>ethnicity (Self reported, ethnicity was classified as: White Americans, African Americans, Asian/native Hawaiian/other Pacific Islander, Indian Americans/Alaskan, Mixed, Unknown)<br>education (Self reported, Years of education completed: <11 = 11; 12; 13; 14; 15; 16; 17+ = 17)
Population characteristics	All participants in the Young Healthy Adults HCP cohort are between 22 and 35 years old, an age range chosen to represent healthy adults beyond the age of major neurodevelopmental changes and before the onset of neurodegenerative changes. 400 participants from the pool of 1206 3T participants were chosen for this study, and 98 participants from the pool of 184 7T participants. The demographics are as follows: First 200 3T participants (n=200; mean age 29; age range 22-36; 108 females, 92 males), second replication dataset of 200 3T participants (n=200; mean age 28; age range 22-36; 99 females, 101 males); third replication dataset of 98 7T participants, i.e. all 7T-HCP young adult participants not already included in either of the 3T datasets and with full resting-state and behavioural data (mean age 29; age range 23-36; 59 females, 39 males). All participants gave informed consent and were reimbursed for their time ($450 for 3T MRI + interview, $400 for 7T MRI) and travel.
Recruitment	No participants were recruited as part of this study.
Ethics oversight	Fully described in the core HCP literature referenced in the manuscript. The paper is only using publicly available datasets.

Note that full information on the approval of the study protocol must also be provided in the manuscript.

Field-specific reporting

Please select the one below that is the best fit for your research. If you are not sure, read the appropriate sections before making your selection.

☐ Life sciences ☒ Behavioural & social sciences ☐ Ecological, evolutionary & environmental sciences

For a reference copy of the document with all sections, see nature.com/documents/nr-reporting-summary-flat.pdf

Behavioural & social sciences study design

All studies must disclose on these points even when the disclosure is negative.

Study description	Data are quantitative experimental data. Participants took part in a two day 3T MRI+behavioural visit and a 2 hour phone interview. Some participants participated in a 7T MRI visit. This manuscript focuses on resting-state fMRI data (from 3T and 7T visits) and questionnaire scores.
Research sample	Our rationale for the research sample was that the HCP-YA dataset is currently the best resting-state fMRI dataset in terms of image quality and volume. Participants had physiological noise recording, which was used for additional data cleanup. Moreover, HCP data collection team made sustainable effort to select participants towards a representational sample. 400 out of the available 1206 3T young healthy adult HCP datasets were used; 98 out of 184 7T young healthy adult HCP datasets were used (see www.humanconnectome.org). Originally we selected participants for balanced mental health scores within the generally healthy sample.

	First, an initial dataset of n=200 (n=200; mean age 29 +/- .26; age range 22-36; 108 females, 92 males) and a second (replication) dataset of the same size as the first (n=200; mean age 28 +/- .28; age range 22-36; 99 females, 101 males) was selected out of the full set of n=1206 3T subjects from the HCP young adults data set (see Klein-Fluegge et al., Nat Hum Beh, 2022). A third dataset contained all 7T-HCP young adult participants not already included in either of the 3T datasets and with full resting-state and behavioural data, which left us with n=98 7T-HCP participants (mean age 29 +/- .33; age range 23-36; 59 females, 39 males; Supplementary Table 1). Following outlier exclusion (specified below), the final dataset included 398 3T and 97 non-overlapping 7T participants. Thus, the manuscript includes a total of n=495 participants which can be considered a large sample in the context of neuroimaging, and representative for a healthy young-adult population.
Sampling strategy	3T participants were chosen from the full HCP data set (https://www.humanconnectome.org/) based on two criteria described in detail in Klein-Fluegge et al., Nat Hum Beh, (2022): the quality of the physiological variables acquired (both cardiac and respiratory; inspected visually and using summary measures such as their variance over time) and their total DSM/ASR scores to allow us to maximise subclinical variance across participants. 7T participants included all available individuals not already included in the 3T sample. The order of magnitude is in thsi study larger then most neuroimaging studies, therefore we have sufficient power for the type of analysis conducted. The sample size was determined based on practical considerations such as full set of measures variables, quality noise recordings and behavioural measures.
Data collection	No data was collected as part of this study. All data acquisition protocols have been published as part of the Human Connectome Project initiative (www.humanconnectome.org) and are summarized in recent publications (e.g., https://www.ncbi.nlm.nih.gov/pubmed/22366334). In the present study, we focused exclusively on the resting-state (rs-) fMRI component of the HCP data as well as recordings of respiration and heart rate. To recap, as part of the HCP, four resting state runs were acquired on a 3T Siemens Skyra 3T scanner or a 7T Siemens Magnetom scanner using custom pulse sequences. 3T resting-state runs lasted 14.4 minutes, had a TR of 720ms, TE of 33ms, isotropic resolution of 2mm, 72 slices, and a multiband factor of 8 resulting in 1200 timepoints. 7T resting-state runs lasted 16 minutes, with a TR of 1 s, TE of 22.2 ms, isotropic resolution of 1.6mm, 85 slices, a multiband factor of 5 and in-plane acceleration factor (iPAT) of 2, resulting in 900 timepoints. Spin-echo images and T1-weighted images were acquired for distortion correction and registration. Cardiac and respiratory signals, were recorded using a pulse oximeter and respiratory bellows fitted to participants prior to the fMRI sessions. Those signals were recorded along with the sync pulse from the scanner at a sampling rate of 400 Hz.
Timing	In this study no data was collected. Already available data was used from the Human Connectome Project (HCP) young adult dataset, here data collection started data in 2012 and the relevant release of the 1200 participants occurred in March 2017. These data were collected between 2012 and 2017 (see Elam et al., Neuroimage, 2021 - The Human Connectome Project: A retrospective).
Data exclusions	Outlier participants from the original pool of 400 3T and 98 (non-overlapping) 7T participants were conservatively rejected based on their individual FC values if more than 10% of their FC values across all edges deviated more than 3.5 standard deviations from the mean across participants. This identified two 3T and one 7T participants as outliers and all analyses were performed on the remaining 398 and 97 participants. Thus, the manuscript includes a total of n=495 participants.
Non-participation	No new participants were recruited as part of this study (all participants were part of the Human Connectome Project initiative as specified above) - so this field does not apply.
Randomization	The experimental design does not involve allocation of participants into different groups. However, predictions were always performed out-of-sample by estimating regression coefficients based on the first half of all 3T participants and applying them to the functional connectivity of the second half of the 3T participants to predict participant's stress scores. We also tested stress predictions in 7T participants using n=398 3T participants' regression coefficients (see supplementary results).

Reporting for specific materials, systems and methods

We require information from authors about some types of materials, experimental systems and methods used in many studies. Here, indicate whether each material, system or method listed is relevant to your study. If you are not sure if a list item applies to your research, read the appropriate section before selecting a response.

Materials & experimental systems

n/a	Involved in the study
x	Antibodies
x	Eukaryotic cell lines
x	Palaeontology and archaeology
x	Animals and other organisms
x	Clinical data
x	Dual use research of concern
x	Plants

Methods

n/a	Involved in the study
x	ChIP-seq
x	Flow cytometry
	x MRI-based neuroimaging

Plants

Seed stocks	N/A
Novel plant genotypes	N/A
Authentication	N/A

Magnetic resonance imaging

Experimental design

Design type	Resting-state fMRI
Design specifications	Four resting state runs were acquired on a Siemens Skyra 3T scanner using custom pulse sequences. Each resting-state run lasted 14.4 minutes.
Behavioral performance measures	No behavioural task was performed during the resting-state acquisition.

Acquisition

Imaging type(s)	functional and structural
Field strength	3T and 7T
Sequence & imaging parameters	3T resting-state runs lasted 14.4 minutes, had a TR of 720ms, TE of 33ms, isotropic resolution of 2mm, 72 slices, and a multiband factor of 8 resulting in 1200 timepoints. 7T resting-state runs lasted 16 minutes, with a TR of 1 s, TE of 22.2 ms, isotropic resolution of 1.6mm, 85 slices, a multiband factor of 5 and in-plane acceleration factor (iPAT) of 2, resulting in 900 timepoints. Spin-echo images and T1-weighted images were acquired for distortion correction and registration (for more details see HCP references in the manuscript).
Area of acquisition	Whole brain

Diffusion MRI ☐ Used ☒ Not used

Preprocessing

Preprocessing software	FSL (e.g., PNM toolbox, FSLnets), Freesurfer (version 5.2), MIGP for group-PCA (Smith et al., NeuroImage, 2014), Connectome Workbench command-line functions and custom-written MATLAB (R2021a), R (version 4.2.1) and BASH scripts.
Normalization	We downloaded the minimally pre-processed HCP data which is described in detail in reference Smith et al., Neuroimage, 2013 (see reference section). In brief, these data are distortion-corrected, temporally filtered, projected on to a surface reconstruction obtained from the T1-weighted image while maintaining subcortical voxels (cifti format), and minimally smoothed. Registration across participants was achieved using multi-modal areal-feature-based surface registration (MSMall, see reference section: Glasser et al., Nature, 2016).
Normalization template	N/A
Noise and artifact removal	Because noise caused by physiological artefacts (e.g. breathing, pulse) is particularly pronounced in brainstem and temporal lobe structures, all key areas for this study, we performed corrections for physiological noise in the data. Removal of artefacts caused by physiological signals is not currently incorporated in standard HCP pipelines. We used the PNM toolbox (https://fsl.fmrib.ox.ac.uk/fsl/fslwiki/PNM; 71) to generate physiological regressors (a total of 33 regressors comprised of: cosine and sine of basic cardiac and respiratory regressors modelled with an order of 4, and thus 16 regressors; multiplicative cardiac and respiratory terms cos(c+r), sin(c+r), cos(c-r), sin(c-r), each modelled using an order of two, and thus again 16 regressors; plus respiration volume per time (RVT)71). In addition to physiological regressors, we constructed 24 motion regressors from the six motion regressors provided (in the HCP data release, these are stored in Movement_Regressors.txt) (e.g., 70): the six original regressors, their derivatives, and the square of the resulting twelve regressors. We also used independent component analysis (ICA)-denoising as provided with the 'fixextended' HCP dataset (melodix_mix and Noise.txt). The motion, physiological and ICA noise regressors were normalized, high-pass filtered and detrended to mimic the pre-processing performed on the data. Then, motion and physiological confounds were aggressively regressed out of the data and ICA components (thus entirely removing any variance explained by physiological or motion parameters), and the noise ICA components were subsequently removed from the data using a soft regression (thus removing only the variance unique to the ICA noise components).

Volume censoring | None

Statistical modeling & inference

Model type and settings

A group average timeseries was generated from the 200 initial 3T data sets using the algorithm MIGP (Smith et al., Neuroimage, 2014, see Reference section). MIGP is a computationally tractable method to approximate the group average time series using group-level PCA. The two parameters specifying (a) the number of data-points kept on-line during the iterative computation of the average and (b) the cut-off describing the number of principal components kept at the end were both set to 4800, corresponding to the number of data points in each individual's file. A dense connectome was created from the average time series using the function cifti-correlation (using Fisher's z). Ringing artefacts were corrected using Wishart RollOff (Glasser et al., Nature, 2016).

Effect(s) tested

No standard fMRI higher-order statistical tests were performed on the group data as usually done with fMRI. Instead, for each participant, we extracted their individual functional connectivity values between hypothalamus nuclei and a priori regions of interest. All tests related to the accuracy (Pearson's correlation coefficient r) with which resting-state functional connectivity could predict a dimensional markers of stress. All predictions were estimated on one half of the training (3T) and applied to the second half of a testing (3T) dataset and therefore generated out-of-sample. For details, please refer to the Methods section.

Specify type of analysis: ☐ Whole brain ☒ ROI-based ☐ Both

Anatomical location(s): All ROIs were determined a priori as explained in the Methods.

Statistic type for inference (See Eklund et al. 2016)

We did not perform any tests across the whole-brain.

Correction

We constructed appropriate null distributions for all tests reported in the manuscript. This always involves repeating the same test n=10,000 times but using a randomized order of participant behavioral scores. Each test incorporates appropriate correction for multiple comparisons. For example, multiple comparison across the 105 models estimated for Figure 3 were performed by only reporting and building a null distribution over the peak prediction, not each individual prediction. Please refer to the Methods section for details.

Models & analysis

n/a	Involved in the study
☐	☒ Functional and/or effective connectivity
☒	☐ Graph analysis
☒	☐ Multivariate modeling or predictive analysis

Functional and/or effective connectivity | Pearson correlation (see Methods)
